# Extracellular vesicles from human semen induce unique tolerogenic phenotypes in vaginal dendritic cells and regulatory T lymphocytes

**DOI:** 10.3389/fimmu.2025.1564002

**Published:** 2025-05-12

**Authors:** Shahrokh Paktinat, Michael G. Gravett, Cara Tobey, Anna Kirby, Whitney Horner, Rebecca Shaffer, Michael Fialkow, Nam Phuong Nguyen, Germán G. Gornalusse, Maryam Kalatehjari, Sean M. Hughes, Florian Hladik, Lucia Vojtech

**Affiliations:** ^1^ Department of Obstetrics and Gynecology, University of Washington, Seattle, WA, United States; ^2^ Molecular Engineering and Sciences Institute, University of Washington, Seattle, WA, United States; ^3^ Department of Medicine, University of Washington, Seattle, WA, United States; ^4^ Vaccine and Infectious Disease Division, Fred Hutchinson Cancer Center, Seattle, WA, United States

**Keywords:** pregnancy, semen, tolerance, extracellular vesicle, dendritic cells, regulatory T cell

## Abstract

**Introduction:**

The regulation of immune responses to promote tolerance to the fetus is critical for successful pregnancy. An understudied aspect of this process is the initiation of regulation pre-conception via exposure to semen. Our study aimed to understand how semen impacts recipient dendritic cells (DCs) and their subsequent role in shaping CD4 T cell differentiation.

**Methods:**

Monocyte-derived DCs (MoDCs) were exposed to semen extracellular vesicles (SEV) or vesicle-depleted semen plasma (VDSP). Phenotypic and functional markers were analyzed using flow cytometry. We also exposed epithelial sheets from vaginal tissue to SEV and VDSP, and measured the number and marker expression of emigrating cells. Finally, we tested how SEV- or VDSP-exposed DCs altered CD4 T cell differentiation by co-culturing exposed MoDCs or tissue emigrated cells with autologous naïve CD4 T cells.

**Results:**

MoDCs exhibited a significant increase of CD141, CD1a, CD38, and ILT4 expression when exposed to SEV or VDSP. A unique feature of semen-treated MoDCs was expression of indoleamine 2,3-dioxygenase (IDO), a potent contributor to the induction of regulatory T cells (Tregs). SEV but not VDSP significantly increased the emigration of intraepithelial DCs. Additionally, SEV significantly enhanced the expression of multiple immunoregulatory markers in the emigrated DCs. After co-culture, we observed significantly more FOXP3+ Tregs expressing high levels of TIGIT in the groups that were initially exposed to SEV.

**Discussion:**

These findings indicate that exposure to SEV induces a tolerogenic program in DCs that can direct differentiation of a unique memory Treg subset, primed for expansion and presumably destined to support a successful pregnancy.

## Introduction

An intricate coordination of the immune system during pregnancy is essential because of the necessity of tolerating the semi-allogeneic fetus. At the fetal-decidua interface, antigen-presenting cells (APCs) interact with trophoblasts and promote tolerance in the adaptive immune system by skewing T cell differentiation towards regulatory phenotypes (1). The decidua is the site of allorecognition during pregnancy (2). However, it is well-described that the priming of the immune system starts prior to embryo implantation, at the time of intercourse. This priming occurs in the cervicovaginal microenvironment where resident immune cells encounter non-self antigens introduced by semen (3–6).

Dendritic cells (DCs) exhibit plasticity in response to various stimuli, enabling the development of distinct functional states. Because they are potent regulators of T cell responses, DCs strongly influence the differentiation and function of T cells (7, 8). For instance, tolerogenic DCs, also known as DC-10, promote the generation of regulatory T cells (Tregs) (9). Tregs can be long lived and are indispensable for maintaining immune tolerance (10). In the context of pregnancy, three major Treg subtypes have been identified in the decidua: CD25^Hi^ FOXP3+ Tregs, PD-1^Hi^ Tregs, and FOXP3+ TIGIT+ Tregs (11). A wide range of inflammatory responses can be precisely controlled within the placental milieu through these three Treg types (11). Studies in mice have highlighted the role of semen fluid, in the absence of sperm, in modulating the induction and functionality of FOXP3+ Tregs in the recipient genital tract (12–14). However, the precise mechanisms by which semen exerts its effects remain to be fully elucidated, especially in humans.

Semen is a complex biofluid that, in addition to sperm, contains bioactive signals which can prime the immune response to a future pregnancy (15, 16). Soluble molecules such as transforming growth factor beta (TGF-β), prostaglandins, many cytokines, and interleukins such as IL-1α, IL-6, and IL-8 are present in semen and likely to affect the vaginal immune microenvironment (17–20). Semen also contains extracellular vesicles (EV) that contain potentially immunoregulatory components, including microRNAs, small non-coding RNAs and cytokines (21–23). Importantly, EV also carry allogeneic major histocompatibility complex (MHC) molecules and potentially other alloantigens, many of which would be expressed by any resulting conceptus (24, 25). Limited research has investigated the immune reactivity of isolated semen EV (SEV) as membrane-enclosed carriers of signals to adaptive immune cells in the recipient (26, 27). Indeed, a persistent question is whether signals delivered via SEV can elicit a tolerance response to co-delivered alloantigens in humans.

Here we interrogated the role of human semen in modulating DC function using an extensive phenotypic and functional characterization of monocyte-derived and vaginal DCs following exposure to SEV and vesicle-depleted semen plasma (VDSP). In addition, we investigated how those SEV- or VDSP-exposed DCs cause differentiation of specific Treg subtypes. We found that SEV, similar to VDSP, induce a unique DC profile marked primarily by induction of indoleamine 2,3-dioxygenase (IDO) and high expression of immunoglobulin-like transcript (ILT4). In vaginal tissue, SEV but not VDSP induced a greater migration of DCs out of epithelial sheets, suggesting that semen EV direct mucosal DC migration to draining lymphatics. When co-cultured with autologous T cells, both monocyte-derived dendritic cells (MoDCs) and mucosal emigrant DCs promoted greater differentiation of FOXP3+ Tregs, particularly those expressing T cell immunoreceptor with Ig and ITIM domains (TIGIT). These results suggest that exposure to semen EV may establish a memory Treg subset poised to expand and contribute to tolerance of the fetus. This memory Treg population may begin to form prior to conception, setting the stage for a successful pregnancy.

## Materials and methods

### Ethical consideration

Written informed consent was provided by the participants. The study’s protocol received approval from the University of Washington’s Institutional Review Boards, under the reference number STUDY00011897, and Fred Hutchinson Cancer Center’s Institutional Review Boards, under the reference number 4323.

### Collection of human blood and semen fluid

Whole blood specimens in acid citrate dextrose solution A (ACD-A) tubes were procured from Bloodworks Northwest (BWNW) Research Institute (Seattle, WA) from healthy donors who self-described as female aged 20-65, and were used to isolate peripheral blood mononuclear cells (PBMCs) within 3–4 hours. Semen samples were obtained from individuals aged 20–65 with normal reproductive health. Semen was collected by masturbation and immediately placed into an aseptic container, and transferred to the laboratory within one hour, then preserved in 4-8°C until processing within 3–4 hours. Due to the viscous nature of semen and for ease of processing, 3 mL of RPMI (Gibco) medium was added to the liquefied semen. Semen plasma was separated from cellular components via centrifugation, and cell debris was filtered out using 0.45 μm syringe filters (Millex HA). Finally, the semen plasma was stored at -80°C until further processing.

### Isolation of PBMCs

Peripheral blood mononuclear cells (PBMCs) were isolated by layering fresh whole blood over Lymphoprep™ density gradient medium in SepMate™ tubes (STEMCELL Technologies), following the manufacturer’s recommended settings. The PBMC fraction was then carefully transferred into a fresh tube and washed with Dulbecco’s phosphate-buffered saline (DPBS; Gibco) prior to counting and viability assessment. The cells were finally cryopreserved in freezing medium containing 10% dimethyl sulfoxide (DMSO; Sigma-Aldrich) in fetal bovine serum (FBS; Peak Serum) for future use.

### Isolation and quantification of semen extracellular vesicles

Semen extracellular vesicles (SEV) were isolated from semen plasma as in previous research (27). In brief, for each batch, semen plasma from five different donors were pooled and layered over a cushion of 30% Sucrose (MP Biomedicals) in 20mM Tris in deuterium oxide (Acros Organics) at pH 7.4 and ultracentrifugation was performed at 100,000g for 90 minutes at 4°C in a Beckman L8-70M ultracentrifuge. The supernatant was collected and layered over a cushion of 25% sucrose in 20mM Tris in deuterium oxide at pH 7.4 for a second ultracentrifugation at 100,000g for 14 hours at 4°C. Afterwards, the supernatant fraction was collected and preserved at -80°C to be used as vesicle-depleted semen plasma (VDSP). The sucrose cushions, containing EV, from both rounds were collected carefully without disturbing pelleted material, and pooled. After rinsing with 15 mL of DPBS, this EV-high suspension was concentrated using an Amicon Ultracel 100 kDa cellulose centrifugal filter to a final volume ranging from 500-1500 μl, and stored at -80°C. The concentration and size distribution of EV were determined using nanoparticle tracking analysis with a Nanosight NS300 instrument (Malvern), following the manufacturer’s instructions. For this purpose, EV samples were vortexed, diluted between 1:6000 and 1:8000 in filtered molecular grade water, and analyzed. Analysis for each sample was performed for 60 seconds using the automatic settings of the Nanosight, and the procedure was repeated five times to derive average concentration values.

### Generation of monocyte-derived DCs

Cryopreserved PBMCs were thawed and washed in RPMI medium completed with 10% heat-inactivated FBS and 1% Penicillin-Streptomycin (Gibco). CD14+ monocytes were isolated using the EasySep™ Human Monocyte Isolation Kit (STEMCELL Technologies). Purified monocytes were then seeded in Costar^®^ cell culture plates (Corning) and differentiated into immature DCs over 5 days using ImmunoCult™ Dendritic Cell Culture Kit (STEMCELL Technologies) with a media change on day 3 based on the manufacturer’s protocol. On day 5, non-adherent DCs were collected by washing the wells, and the adherent DCs were detached after a 20-minute incubation in DPBS containing 2.5 mM Ethylenediaminetetraacetic acid (EDTA). To obtain IL-10-generated tolerogenic DCs, 10 ng/mL of IL-10 (Peprotech) was added to cultures at day 0 and day 3.

### Characterizing SEV-exposed MoDCs by high-parameter flow cytometry

To characterize the response of DCs to vesicular and non-vesicular fractions of semen plasma, immature monocyte-derived DCs (MoDCs) were exposed to either SEV or VDSP overnight. SEV were added to the DCs at a concentration of one million vesicles per cell and VDSP was used at a concentration of 5% volume/volume, both in 100 uL AIM V™ serum free medium (Gibco) in a round-bottom 96-well plate (Corning). The chosen concentrations were based on previous studies (27, 28). DPBS served as the background control, and Interleukin-10 (IL-10)-generated tolerogenic DCs were used as a positive control for tolerogenic status. Following the incubation, the cells were stained for high-parameter analysis, utilizing an established flow cytometry panel (29), with some modifications (**Supplementary Table 1**), to enable detailed cellular characterization of the human DCs. Flow cytometry was performed using a BD FACSymphony™ A3, and voltages were calibrated prior to each run using SPHERO™ Ultra Rainbow Calibration Particles (Spherotech), at the UW Cell Analysis Facility.

### Exposing vaginal epithelial sheets to semen components

Vaginal tissue samples were obtained from repair surgeries at the University of Washington Medical Center. These surgeries were not done for cancer treatment reasons and the patients were not taking androgenizing hormones, but we do not have any other demographic details for these samples. The stromal layer was carefully excised, and precise punch biopsies of 5 mm diameter were taken from the tissue. The biopsies were then incubated in a solution of dispase II enzyme (Sigma-Aldrich) at a concentration of 12.5 IU/mL in Hank’s Balanced Salt Solution (HBSS) containing calcium and magnesium for 2 hours at 37°C. Following incubation, the epithelial sheets were peeled off and thoroughly washed in DPBS.

To assess the migratory behavior of intraepithelial immune cells upon exposure to semen components, the epithelial sheets were placed in hanging cell culture inserts with pore size of 8 µm (Millicell) containing AIM V™ serum free medium (Gibco) supplemented with 1% amphotericin B (Gibco). An equal number of 5 mm diameter epithelial sheets were allocated to the experimental groups, which included a no-treatment control group, a group treated with SEV, and a group treated with VDSP. The next day, cells that had migrated through the inserts into the lower chamber were collected and characterized by flow cytometry (**Supplementary Table 2**) with absolute quantification using cell counting beads (CountBright™ Absolute Counting Beads, Invitrogen). Flow cytometry was performed using a Cytek^®^ Aurora Full Spectrum Flow Cytometry System at the UW Cell Analysis Facility.

### Profiling of regulatory T cells generated in DC-naïve CD4+ T cell co-culture by flow cytometry

The functional responses of SEV-exposed MoDCs and mucosal emigrant cells were assessed using a co-culture system with naïve CD4+ T cells, designed to simulate the immunological interactions in draining lymph nodes. Naïve CD4+ T cells were isolated from autologous PBMCs by EasySep™ Human Naïve CD4+ T Cell Isolation Kit (STEMCELL Technologies). Subsequently, SEV-exposed, VDSP-exposed, or control DCs were co-cultured with purified naïve CD4+ T cells at a ratio of 1:3. We selected a 1:3 DC to naïve CD4^+^ T cell ratio to enhance the likelihood of sustained, productive interactions over a two-week co-culture period without the addition of exogenous cytokines. This moderately dense ratio was chosen to support sufficient T cell stimulation and survival in a cytokine-free environment, where antigen presentation efficiency may be lower, especially given that only a subset of DCs may be activated by SEV or VDSP exposure. While this ratio exceeds physiological DC:T cell ratios found in lymph nodes, supra-physiological ratios are commonly used *in vitro* to compensate for the absence of the complex tissue microenvironment (30–32). In addition, other control groups including naïve CD4+ T cells cultured alone or treated with either SEV or VDSP were used. This was to ascertain the direct effects of these components on the naïve CD4+ T cells, independent of DC interaction. For cultures using mucosal emigrating DCs, variable ratios were employed in mucosal emigrant-naïve CD4+ T cell co-cultures due to the unpredictable yield of emigrated DCs within the emigrant cell population. After 13 days of co-culture, cells were stained for flow cytometry analysis (**Supplementary Table 3**) to detect three subtypes of Tregs, CD25^Hi^ FOXP3+ Tregs, LAG3+ CD49b+ Tr1-like Tregs, and FOXP3+ TIGIT+ Tregs, found in the context of pregnancy. Flow cytometry was performed using a Cytek^®^ Aurora Full Spectrum Flow Cytometry System at the UW Cell Analysis Facility.

### Statistics

Statistical analyses were performed using GraphPad Prism 9, applying two-tailed paired t-tests for comparisons between treatment groups and the control. Significance levels were denoted as follows: *p ≤ 0.05, **p ≤ 0.01, ***p ≤ 0.001, ****p ≤ 0.0001. Any p-value below 0.05 was deemed to indicate statistical significance.

## Results

### SEV and VDSP induce tolerogenic and immunoregulatory marker expression on MoDCs without affecting costimulatory or coinhibitory markers

**Figure 1:**
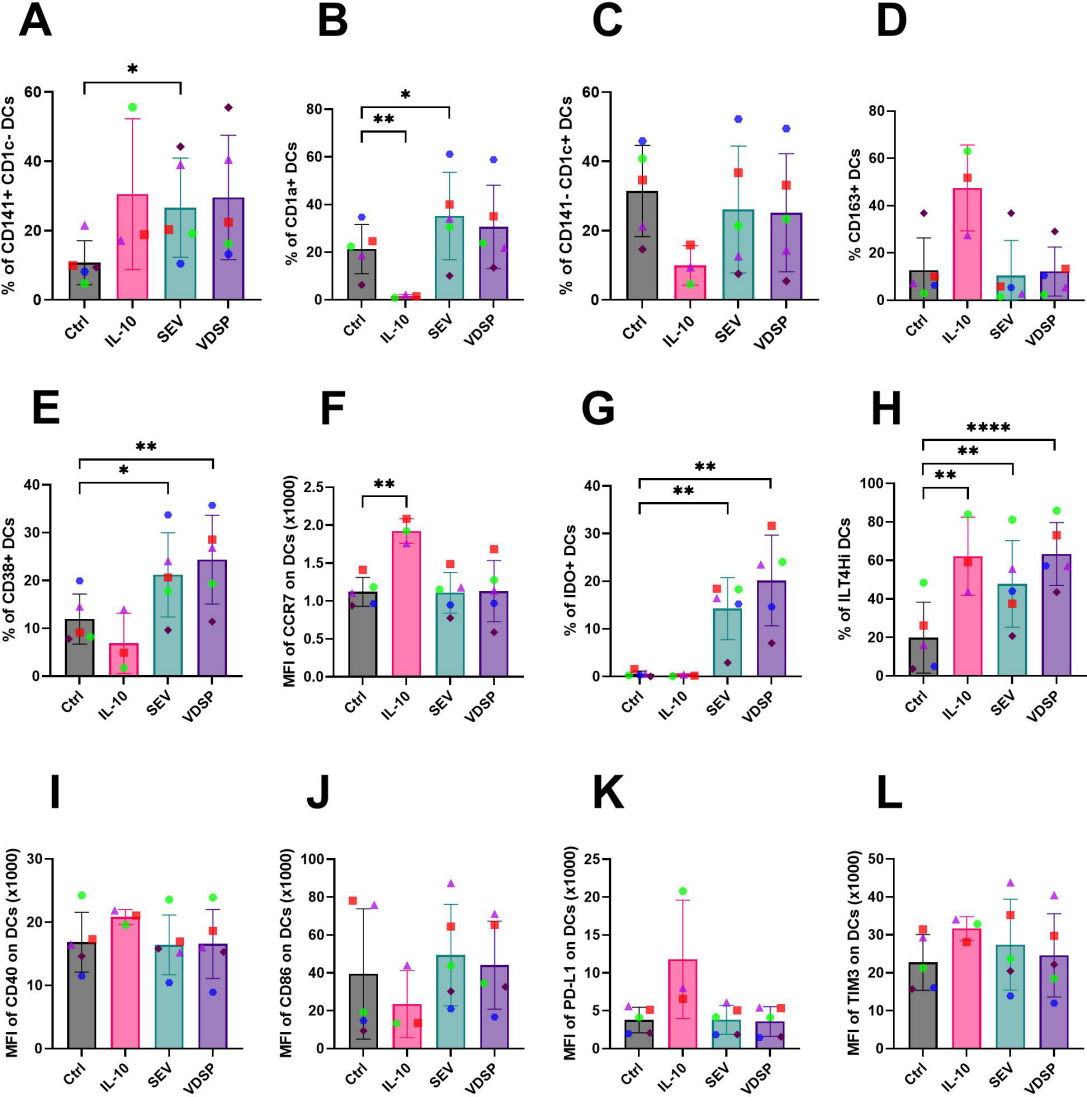
Phenotypic changes induced by SEV and VDSP on monocyte-derived dendritic cells (MoDCs) defined as live CD45+ CD14- CD19- CD3- CD56- CD16- CD11c+ HLA-DR+ cells. **(A)** % CD141+ CD1c-, **(B)** % CD1a+, **(C)** % CD141- CD1c+, **(D)** % CD163+, **(E)** % CD38+, **(F)** median fluorescent intensity (MFI) of CCR7, **(G)** % IDO+, **(H)** % ILT4Hi, **(I)** MFI of CD40, **(J)** MFI of CD86, **(K)** MFI of PD-L1, and **(L)** MFI of TIM3. Each data point represents the average of two independent experiments conducted on the same donor on different days. Paired samples are represented by the same color/shape. Missing data points in the IL-10 group are due to insufficient cell numbers. A two-tailed paired t-test was used to compare treatment groups with the control. Significance levels are indicated as follows: *p ≤ 0.05, **p ≤ 0.01, ****p ≤ 0.0001.

Semen was collected from individuals with uncomplicated reproductive health and semen extracellular vesicles (SEV) and vesicle depleted seminal plasma (VDSP) was isolated by density gradient ultracentrifugation (see methods). Our previous work demonstrated that SEV alter dendritic cell (DC) function and did not directly affect T cells (27). Here our goal was to examine phenotypic and functional changes in DCs induced by SEV and VDSP. Due to the limited availability of tissue-resident dendritic cells (DCs), we initially used monocyte-derived DCs (MoDCs) as an *in vitro* model to investigate the direct effects of semen components on these cells. We employed a flow cytometry panel primarily designed to characterize DC subpopulations. The panel was capable of identifying canonical DC subpopulations, specifically CD141+ CD1c- cross-presenting conventional DC1 (cDC1), CD1c+ CD141- conventional DC2 (cDC2), and CD141- CD1c- (double negative) DCs, as well as CD1a expressing DCs. **Supplementary Figure S1** illustrates the panel resolution. We found phenotypic changes in MoDCs, defined as live CD45+ CD14- CD19- CD3- CD56- CD16- CD11c+ HLA-DR+ cells, following overnight exposure to SEV, marked by a significant rise in CD141+ CD1c- and CD1a+ DCs compared to the control group (**Figures 1A, B**). A similar pattern was observed with VDSP exposure. In contrast, MoDCs treated with IL-10 as a positive control tolerizing stimulus, displayed a distinct phenotype, characterized by nearly undetectable levels of CD1a+ DCs and low CD1c expression (**Figures 1B, C**).

We then analyzed the expression of functional markers in the total MoDC population. We observed no differences in CD16 and CD11b expression in any of the groups (not shown). Treatment with IL-10 caused a marked upregulation of CD163-expressing DCs (**Figure 1D**). We observed a significant upregulation of CD38, a maturation and migration marker, in the SEV and VDSP-exposed groups (**Figure 1E**). We also looked at another migration marker, CCR7. Unlike IL-10 treated MoDCs, which significantly upregulated CCR7, SEV and VDSP treated cells did not change CCR7 expression (**Figure 1F**).

We additionally analyzed the expression of molecules previously linked to tolerogenic functions in APCs. The most striking effect of semen components on MoDCs was the induction of indoleamine 2,3-dioxygenase (IDO), a known contributor to the induction of regulatory T cells (Tregs). IDO was dramatically upregulated following exposure to SEV or VDSP, but not IL-10 (**Figure 1G**). Furthermore, both SEV and VDSP (as well as IL-10) significantly upregulated ILT4 (**Figure 1H**), an HLA-G receptor associated with enhanced differentiation of classical FOXP3+ Tregs (33).

We next analyzed markers that mediate DCs interactions with T cells. We did not observe downregulation of co-stimulatory markers CD40 (**Figure 1I**), CD86 (**Figure 1J**) or CD80 (data not shown) in comparison to the control, as observed in some tolerogenic DCs (34). In 4 out of 5 tested donors SEV slightly increased CD86 expression. No changes were observed in expression levels of PD-L1 (**Figure 1K**) and TIM3 (**Figure 1L**), both of which are coinhibitory markers that suppress T cell activation. In contrast, IL-10 treatment seemed to reduce CD86 expression (**Figure 1J**) and induce PD-L1 expression (**Figure 1K**).

Collectively, these findings demonstrate that SEV and soluble semen components induce a unique phenotype in MoDCs, unlike either untreated or IL-10-treated cells. These cells are distinguished by their maintained co-stimulatory molecules, enhanced antigen-presenting and migratory markers, and upregulation of key tolerogenic molecules such as IDO and ILT4. This suggests these DCs sustain the ability to traffic through the lymphatics and present antigens to T cells, while co-delivering some signals linked to the differentiation of regulatory T cells.

### SEV induce immunoregulatory phenotypes in *ex vivo* vaginal tissue DCs

**Figure 2:**
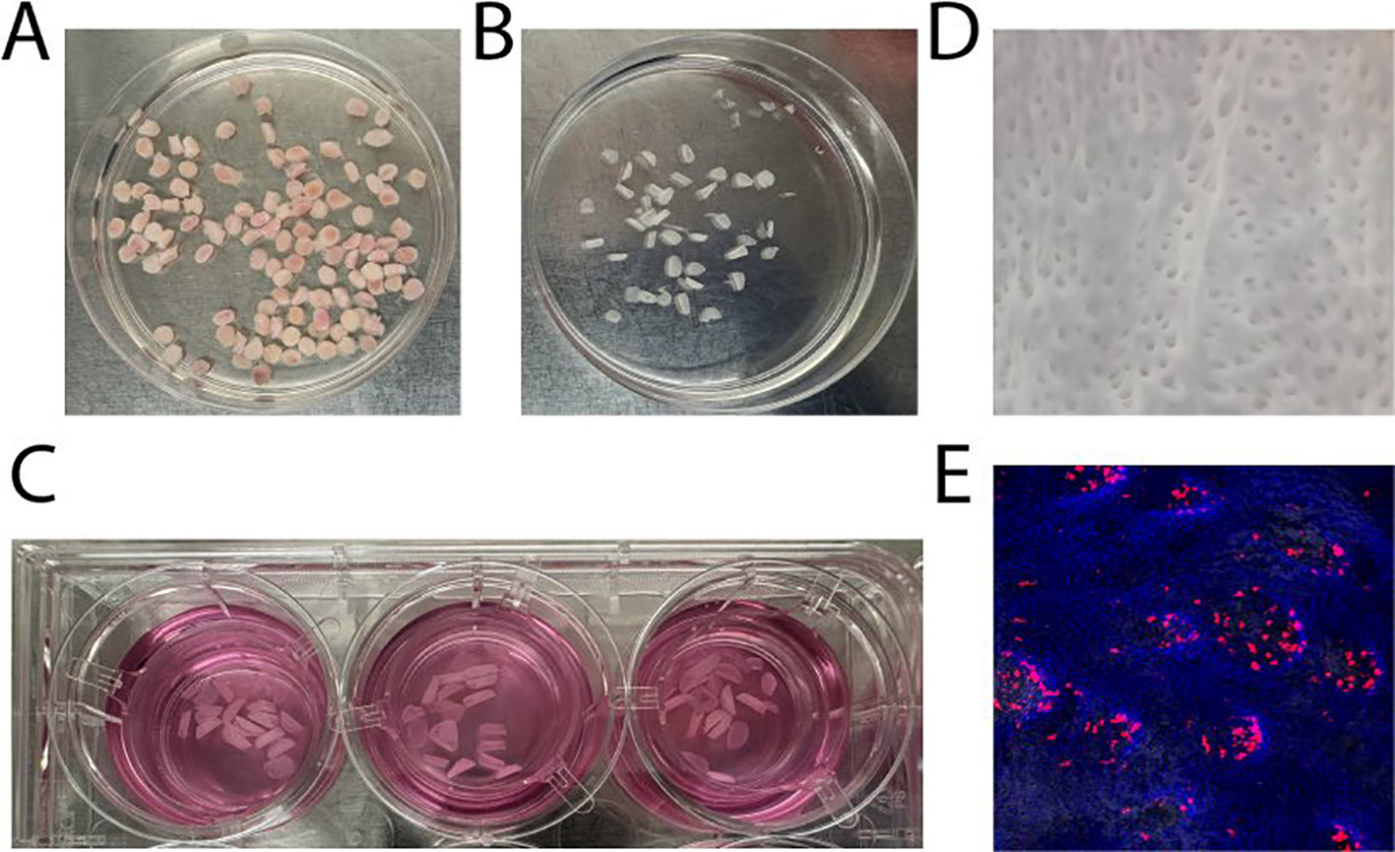
Preparation of epithelial sheets and localization of dendritic cells. **(A)** Biopsies of 5 mm diameter were punched from vaginal tissue samples. **(B)** Dispase treatment was used to carefully peel epithelial sheets from the underlying stromal layer. **(C)** Equal numbers of 5 mm epithelial sheets were exposed to SEV or VDSP fractions in transwell inserts to facilitate the analysis of emigrating cells. **(D)** The stromal-facing surface of an epithelial sheet displays distinct openings, corresponding to regions where stromal papillae were detached during the peeling process. **(E)** Immunofluorescence staining highlights CD1a+ cells (red) positioned adjacent to the papillary regions of an epithelial sheet. Nuclei are stained with DAPI.

To determine whether SEV induce similar changes in tissue-derived cells, we obtained punch biopsies of 5 mm diameter from vaginal tissues (**Figure 2A**), peeled epithelial sheets using dispase (**Figure 2B**), and exposed an equal number of 5 mm diameter epithelial sheets to semen fractions in transwell inserts (**Figure 2C**) to facilitate the analysis of emigrating mucosal cells. DCs which are activated and induced to migrate will tend to move downwards through the transwell, even in the absence of chemoattractants. This approach is similar to one described previously (35–37). **Figure 2D** illustrates the stromal-facing surface of an epithelial sheet, highlighting discrete openings where stromal papillae have been pulled out during the peeling process. **Figure 2E** demonstrates the localization of CD1a+ (red) DCs in close proximity to the papillary regions, consistent with their role in immune surveillance.

**Figure 3:**
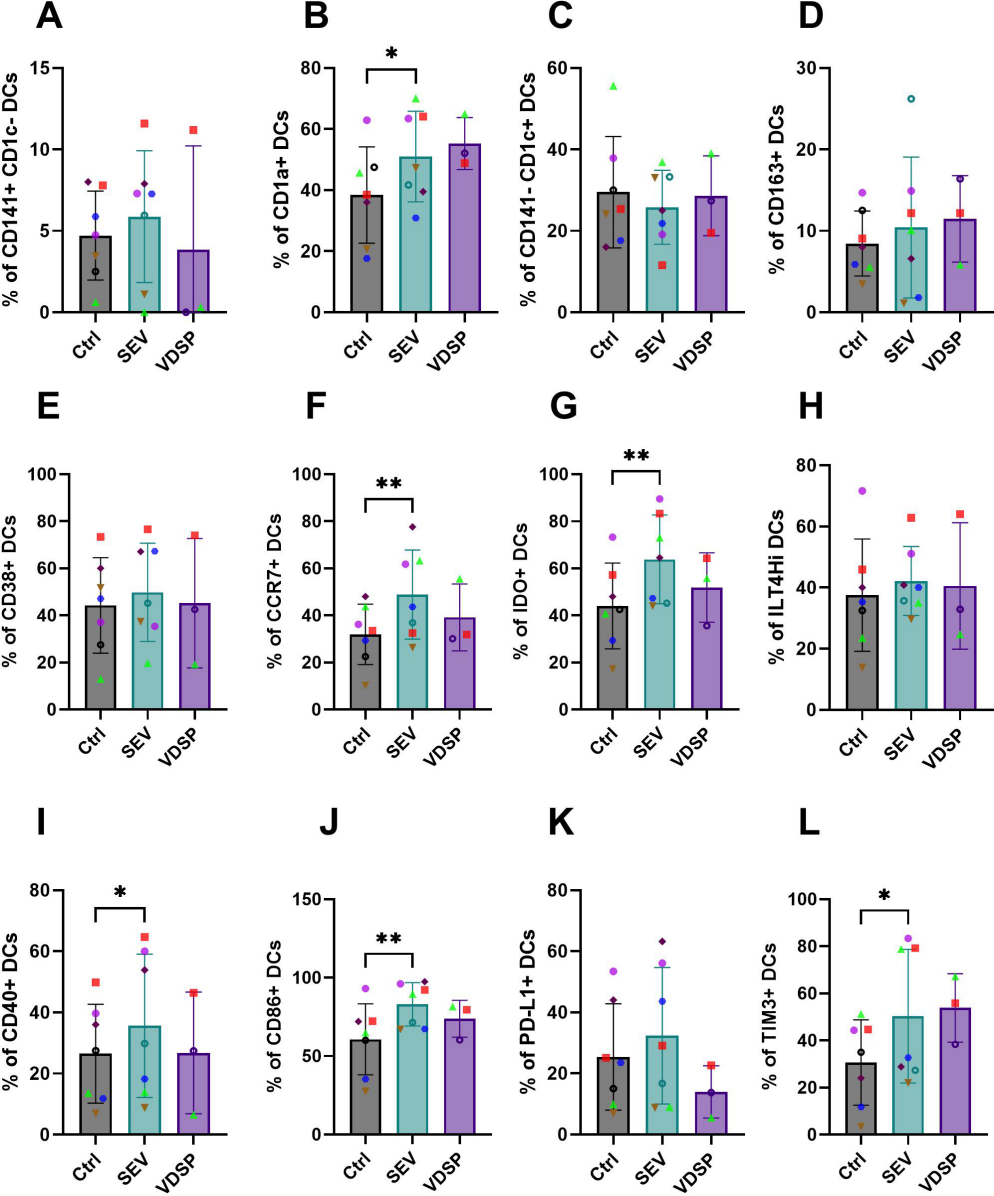
Phenotypic changes induced by SEV and VDSP on live CD45+ CD19- CD56- CD11c+ HLA-DR+ emigrated vaginal dendritic cells (DCs). **(A)** % CD141+ CD1c-, **(B)** % CD1a+, **(C)** % CD141- CD1c+, **(D)** % CD163+, **(E)** % CD38+, **(F)** % CCR7+, **(G)** % IDO+, **(H)** % ILT4Hi, **(I)** % CD40+, **(J)** % CD86+, **(K)** % PD-L1+, and **(L)** % TIM3+. Each data point represents a single donor. Paired samples are represented by the same color/shape. Missing data points in VDSP group are due to insufficient tissue amount. A two-tailed paired t-test was used to compare treatment groups with the control. Significance levels are indicated as follows: *p ≤ 0.05, **p ≤ 0.01.

**Figure 4:**
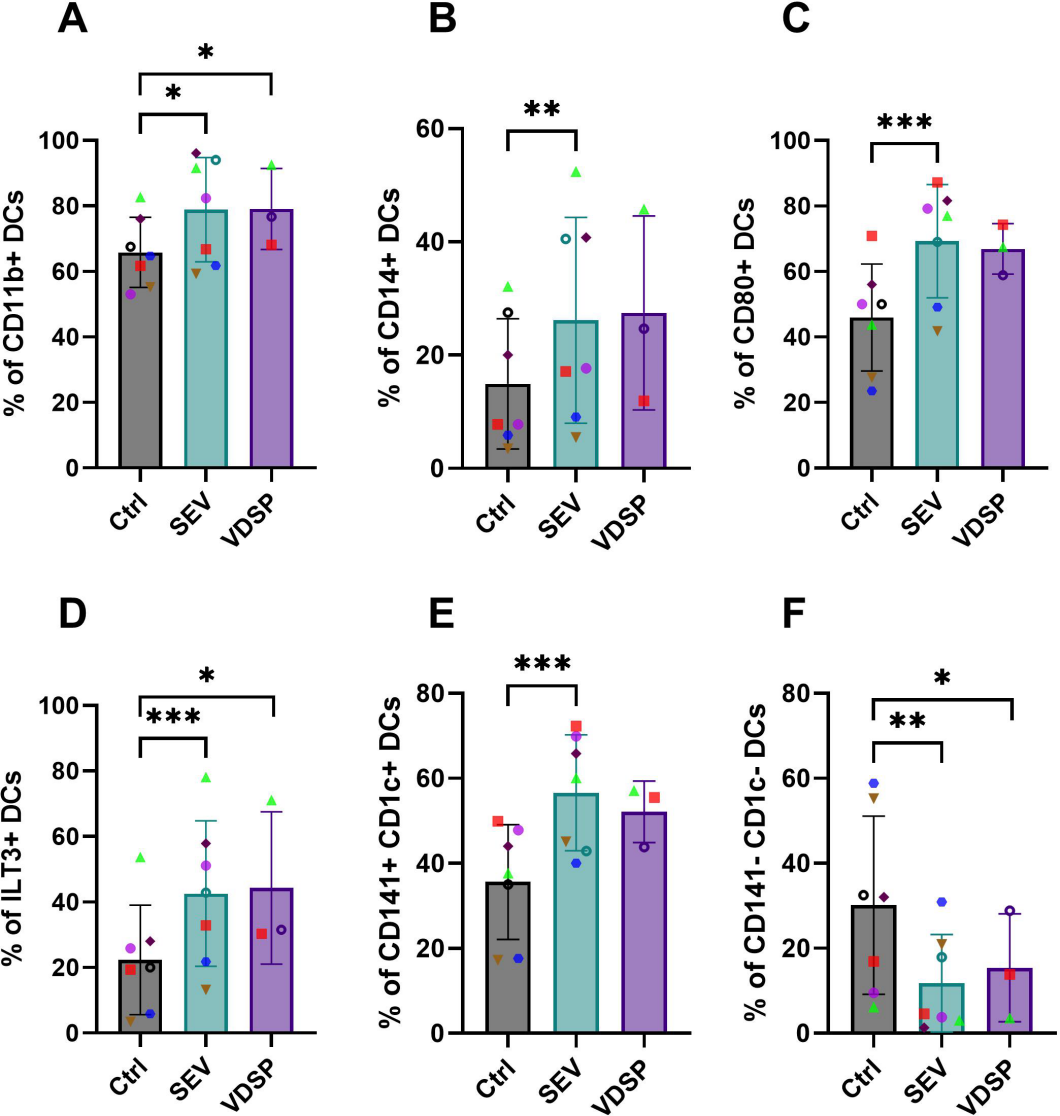
Phenotypic changes induced by SEV and VDSP on live CD45+ CD19- CD56- CD11c+ HLA-DR+ emigrated vaginal dendritic cells (DCs). **(A)** % CD11b+, **(B)** % CD14+, **(C)** % CD80+, **(D)** % ILT3+, **(E)** % CD141+ CD1c+, and **(F)** % CD141- CD1c-. Each data point represents a single donor. Paired samples are represented by the same color/shape. Missing data points in VDSP group are due to insufficient tissue amount. A two-tailed paired t-test was used to compare treatment groups with the control. Significance levels are indicated as follows: *p ≤ 0.05, **p ≤ 0.01, ***p ≤ 0.001.

We analyzed marker expression in emigrated DCs, defined as live CD45+ CD19- CD56- CD11c+ HLA-DR+ cells, in the lower compartment of the transwell, which revealed differences in the SEV-exposed group compared to the control (**Figures 3A–L**). This included significant increased frequencies of CD1a+ (**Figure 3B**), CCR7+ (**Figure 3F**), IDO+ (**Figure 3G**), CD86+ (**Figure 3J**), and TIM3+ (**Figure 3L**) DCs. In addition, tissue emigrant DCs showed significant upregulation in CD11b (**Figure 4A**), CD14 (**Figure 4B**), CD80 (**Figure 4C**), and ILT3 (**Figure 4D**) expression after SEV exposure, and CD11b and ILT3 after VDSP exposure. These changes in CD11b, CD14, CD80 and ILT3 expression were undetectable in SEV- or VDSP-exposed MoDCs. Notably, a higher number of CD141+CD1c+ DCs, which have been suggested to have a strong capacity to home to lymph nodes following vaccination (38), were found among the emigrated DCs in the SEV group (**Figure 4E**). This double-positive population also increases in MoDCs following exposure to SEV, albeit to a lesser extent (**Supplementary Figure S2**). SEV and VDSP significantly reduced the frequency of CD141-CD1c- DCs (**Figure 4F**). This population constitutes a distinct subset within the human myeloid DC compartment enriched for type-I interferon and antiviral responses (39), which may reflect the downregulation of inflammation-responsive DCs by semen to promote a tolerogenic environment. Interestingly, no differences were observed in non-emigrant DCs collected from the top chamber of the transwell system (**Supplementary Figures S3**) following exposure to semen fractions. Taken together, vaginal tissue derived DCs responded to SEV exposure in similar ways to MoDCs, suggesting a high migratory potential and capacity for antigen-presentation and T cell interactions, yet combined with upregulation of tolerogenic molecules.

### SEV induce increased DC migration out of vaginal epithelium

**Figure 5:**
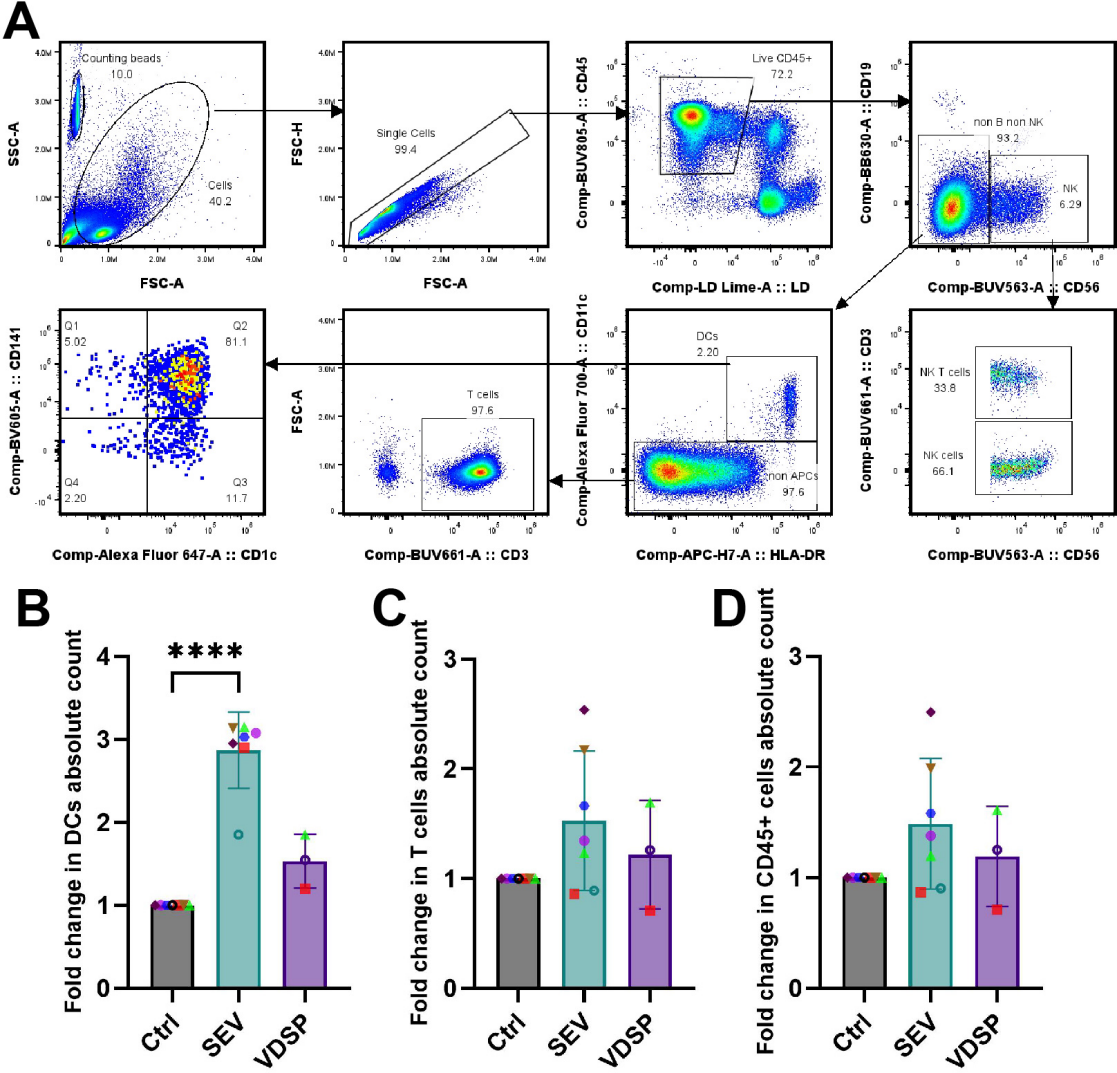
SEV increase the migration of dendritic cells (DCs) out of vaginal epithelium. **(A)** Flow cytometry gating scheme for characterizing vaginal tissue emigrated cells upon exposure to semen extracellular vesicles (SEV) or vesicle-depleted semen plasma (VDSP). DCs were defined as live CD45+ CD19- CD56- CD11c+ HLA-DR+, natural killer (NK) cells were defined as live CD45+ CD19- CD56+, NK T cells were defined as live CD45+ CD19- CD56+ CD3+, and T cells were defined as live CD45+ CD19- CD56- CD11c- CD3+ cells. Phenotyping of DCs was performed by gating on CD141 and CD1c expression. Gating on counting beads was performed to achieve absolute quantification. **(B)** fold change in absolute count of emigrated live CD45+ CD11c+ HLA-DR+ DCs, **(C)** fold change in absolute count of emigrated live CD45+ CD3+ T cells, **(D)** fold change in absolute count of live CD45+ cells. Each data point represents a single donor. Paired samples are represented by the same color/shape. Missing data points in VDSP group are due to insufficient tissue amount. A two-tailed paired t-test was used to compare treatment groups with the control. Significance levels are indicated as follows: ****p ≤ 0.0001.

To assess the direct effect of semen components on the migratory capacity of vaginal epithelial immune cells, we quantified the absolute numbers of various immune cell types that emigrated out of the tissue across the transwell membrane into the bottom well. **Figure 5A** illustrates the gating strategy employed for identifying vaginal immune cell subtypes. SEV specifically increased the migration of DCs into the bottom well (**Figure 5B**), with no such effect observed for other cell types, such as T cells or even total immune cells (**Figures 5C, D**). Interestingly, VDSP showed no impact on cell migration in any population. Thus, SEV but not VDSP cause increased DC migration out of mucosal tissues.

### SEV-exposed DCs induce regulatory T cell subsets and increase activation and inhibitory marker expression on CD4+ T cells

**Figure 6:**
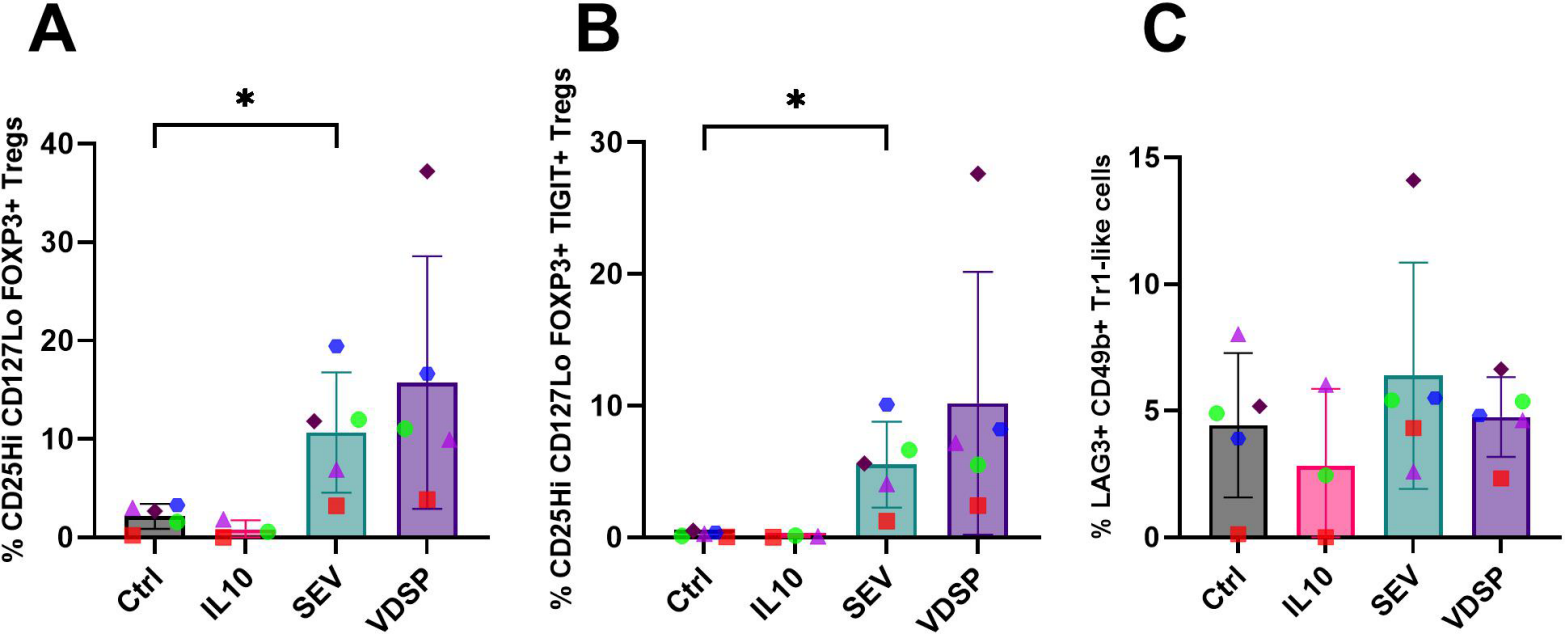
SEV -exposed monocyte-derived dendritic cells (MoDCs) favor the differentiation of FOXP3+ regulatory T cells. **(A)** % CD25Hi CD127Lo FOXP3+ in live CD45+ CD3+ CD4+, **(B)** % CD25Hi CD127Lo FOXP3+ TIGIT+ in live CD45+ CD3+ CD4+, **(C)** % LAG3+ CD49b+ Tr1-like Tregs in live CD45+ CD3+ CD4+. Each data point represents the average of two independent experiments conducted on the same donor on different days. Paired samples are represented by the same color/shape. Missing data points in the IL-10 group are due to insufficient cell numbers. A two-tailed paired t-test was used to compare treatment groups with the control. Significance levels are indicated as follows: *p ≤ 0.05.

In order to determine how semen-exposed DCs affect T cell differentiation, we exposed MoDCs to semen components and then co-cultured these MoDCs with naïve CD45RA+CD45RO- CD4+ T cells from the same MoDC donor. Naïve CD4+ T cells cultured in the absence of DCs, even with SEV or VDSP, did not survive or proliferate well, so further analysis was not performed on these cells. After two weeks of co-culture we analyzed marker expression on CD4+ T cells. We first gated cells based on previously identified subsets of regulatory CD4+ T cells present at the fetal-decidua interface (11), i.e. FOXP3+ Tregs, Tr1-like Tregs, and FOXP3+TIGIT+ Tregs (for gating scheme see **Supplementary Figure S4**). We observed a significant increase in classical CD25Hi CD127Lo FOXP3+ Tregs in co-cultures with SEV-exposed MoDCs (**Figure 6A**). The TIGIT+ Tregs were found exclusively in the SEV and VDSP groups (**Figure 6B**). We did not observe an increase in Tr1-like CD4+ cells co-expressing LAG3 and CD49b (**Figure 6C**). Thus, SEV- or VDSP-exposed MoDCs preferentially induce TIGIT+ Tregs.

**Figure 7:**
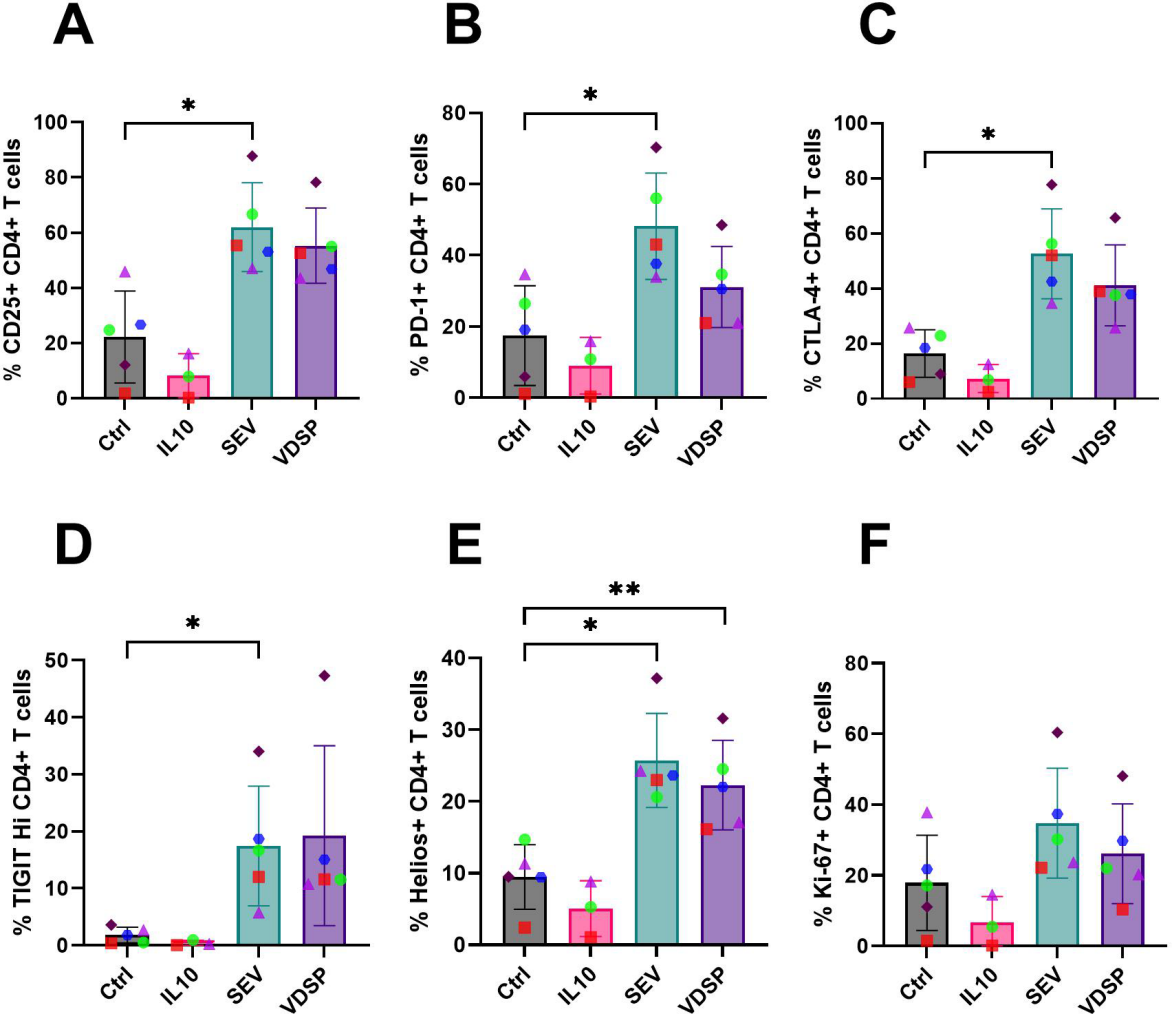
SEV- and VDSP-exposed monocyte-derived dendritic cells (MoDCs) increase activation and inhibitory marker expression on CD4+ T cells. **(A)** % CD25+ in live CD45+ CD3+ CD4+, **(B)** % PD-1+ in live CD45+ CD3+ CD4+, **(C)** % CTLA-4+ in live CD45+ CD3+ CD4+, **(D)** % TIGIT^Hi^ in live CD45+ CD3+ CD4+, **(E)** % Helios+ in live CD45+ CD3+ CD4+, and **(F)** % Ki-67+ in live CD45+ CD3+ CD4+. Each data point represents the average of two independent experiments conducted on the same donor on different days. Paired samples are represented by the same color/shape. Missing data points in the IL-10 group are due to insufficient cell numbers. A two-tailed paired t-test was used to compare treatment groups with the control. Significance levels are indicated as follows: *p ≤ 0.05, **p ≤ 0.01.

We also analyzed total CD4+ T cells for the expression of other regulatory markers (for gating scheme see **Supplementary Figure S4**). SEV-exposed MoDCs induced CD4+ T cells with notably increased expression of regulatory markers, including CD25, PD-1, CTLA-4, and TIGIT (**Figures 7A–D**). This was accompanied by a significant upregulation of Helios (**Figure 7E**), suggested as a marker of human Treg stability (40), and a slight increase in proliferation, as indicated by elevated Ki-67 levels (**Figure 7F**). VDSP-exposed MoDCs only upregulated the expression of Helios in CD4+ T cells (**Figure 7E**).

**Figure 8:**
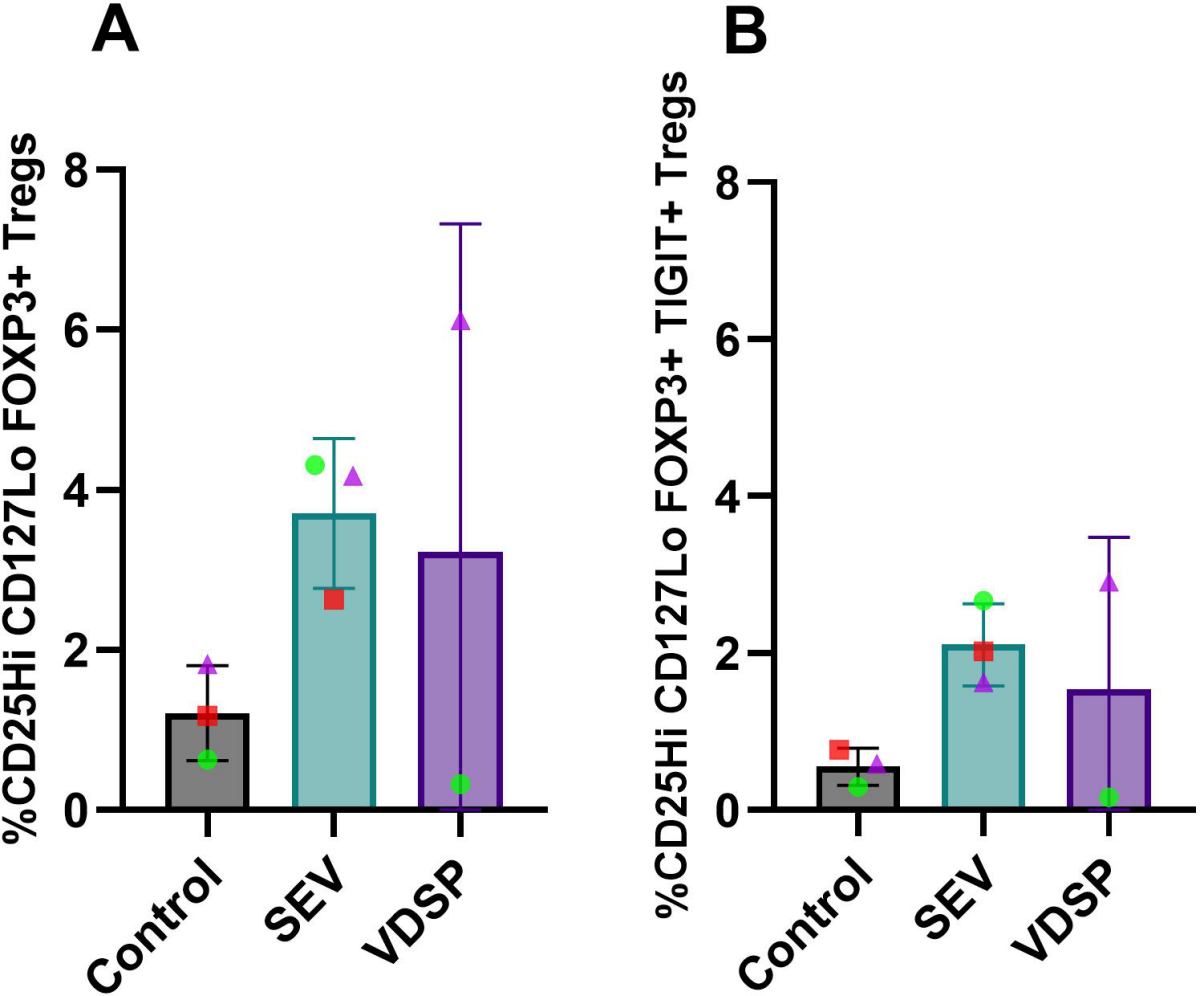
SEV-exposed vaginal emigrant cells favor the differentiation of FOXP3+ regulatory T cells. **(A)** % CD25Hi CD127Lo FOXP3+ in live CD45+ CD3+ CD4+, and **(B)** % CD25Hi CD127Lo FOXP3+ TIGIT+ in live CD45+ CD3+ CD4+. Each data point represents a single donor. Paired samples are represented by the same color/shape. Missing data points in the VDSP group are due to insufficient tissue amount. A two-tailed paired t-test was used to compare treatment groups with the control.

**Figure 9:**
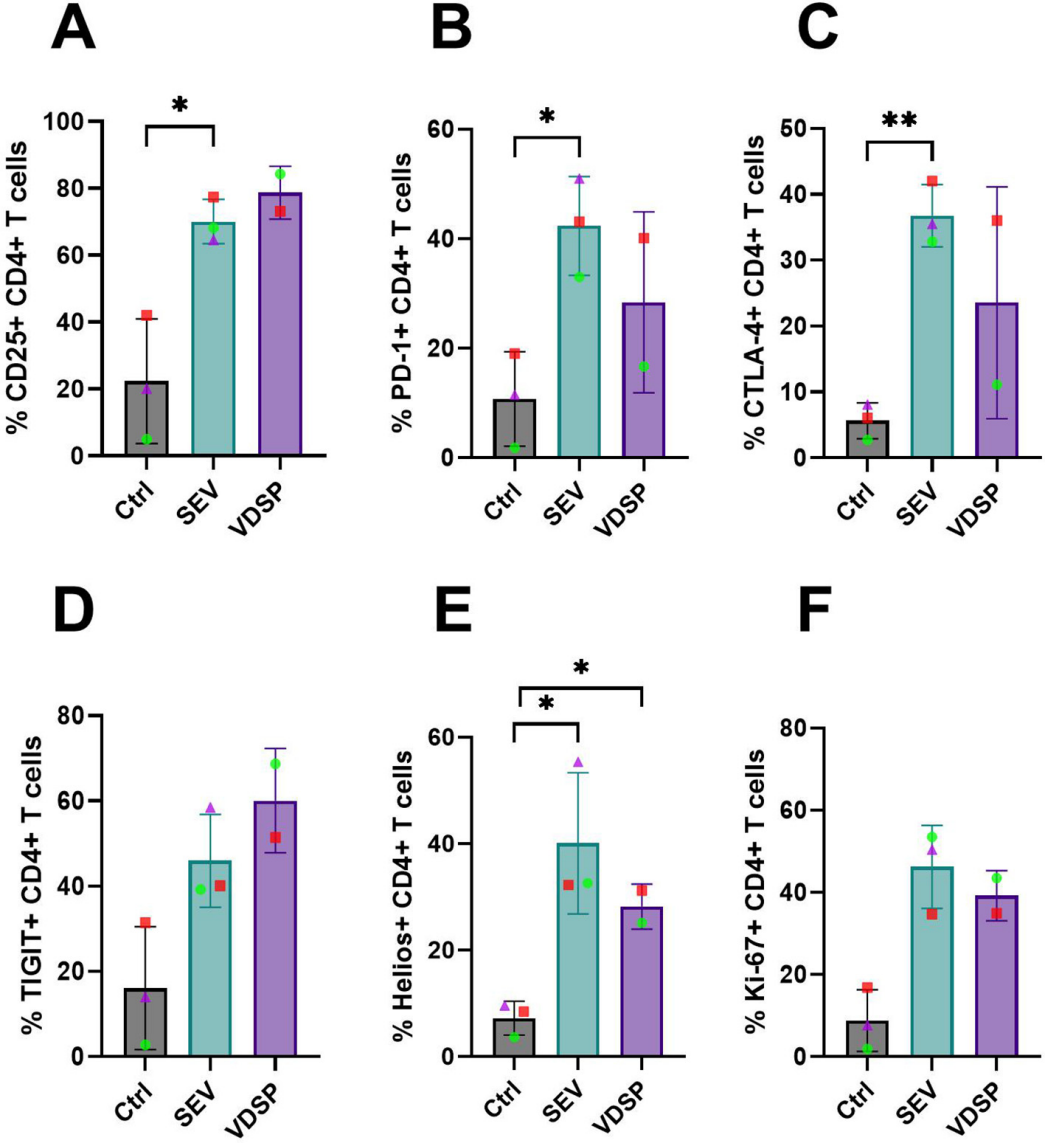
SEV-exposed vaginal emigrant cells increase activation and inhibitory marker expression on CD4+ T cells. **(A)** % CD25+ in live CD45+ CD3+ CD4+, **(B)** % PD-1+ in live CD45+ CD3+ CD4+, **(C)** % CTLA-4+ in live CD45+ CD3+ CD4+, **(D)** % TIGIT+ in live CD45+ CD3+ CD4+, **(E)** % Helios+ in live CD45+ CD3+ CD4+, and **(F)** % Ki-67+ in live CD45+ CD3+ CD4+. Each data point represents a single donor. Paired samples are represented by the same color/shape. Missing data points in the VDSP group are due to insufficient tissue. A two-tailed paired t-test was used to compare treatment groups with the control. Significance levels are indicated as follows: *p ≤ 0.05, **p ≤ 0.01.

We repeated this experiment in tissue-derived cells, co-culturing SEV or VDSP-exposed vaginal tissue emigrant cells with autologous naïve peripheral T cells. Due to very low DC yields from some tissue samples we were not able to include the VDSP condition in some experiments. Given that SEV and VDSP has similar effects on cells for most assays, but SEV usually caused a stronger phenotype, in particular for cell migration, we considered this a more important condition to include. Similar to results with MoDCs (**Figure 6**), we observed increased differentiation of CD4+ T cells into classical FOXP3+ Tregs and a TIGIT+ subpopulation of FOXP3+ Tregs (**Figures 8A, B**), however this increase did not reach significant level in our samples. Additionally, analysis of other regulatory markers, specifically CD25, PD-1, CTLA-4, TIGIT, Helios, and Ki-67, showed a similar trend of increase on CD4+ T cells induced by SEV (**Figures 9A–F**). VDSP-exposed cells only upregulated the expression of Helios in CD4+ T cells (**Figure 9E**).

In summary, the emergence of a Treg population from naïve CD4+ T cells co-cultured with SEV-exposed monocyte-derived or vaginal DCs demonstrates a mechanism whereby contact with a partner’s semen induces a tolerizing environment in the recipient reproductive tract, poised to support a healthy pregnancy (41).

## Discussion

Partner-specific tolerance following exposure to semen is well documented in the mouse model (41–45), but not in humans. In humans, there is no direct evidence of partner-specific Tregs, but epidemiological studies support their existence. Prolonged exposure to a particular partner’s semen through intercourse is associated with a lower risk of pregnancy complications such as preeclampsia, whereas a change in partner has been shown to increase this risk (46–51). Long-lived tolerance to antigens delivered in semen implies memory cells, initially differentiated by antigen-presenting cells (APCs) co-presenting semen antigens alongside tolerance inducing signals (52). In mice, exposure to semen induces APC development, and classical FOXP3+ memory Tregs specific for semen-delivered antigens have been identified (14, 42, 45). However, it is unclear which fractions of semen are important and what tolerance phenotypes are induced. Here, we sought to characterize how exposure to specific cell-free fractions of semen phenotypically and functionally impact APCs and T cells. Importantly, we did this exclusively with human cells and tissues.

Exposure to semen *in vivo* leads to a robust immune response, partly characterized by a rapid influx of leukocytes, including macrophages and CD1a+ DCs, into the genital mucosa (5). Previous studies culturing MoDCs with unfractionated semen showed differentiation of the cells into a regulatory phenotype characterized by low inflammatory cytokine production, impaired maturation, and high IL-10 and TGFB expression. In turn, this MoDC phenotype contributes to the expansion of FOXP3+ regulatory T cells (20, 53). Semen contains a high concentration of immunosuppressive PGE2 and TGFB, as well as many other bioactive molecules (54–56). In addition to soluble factors, semen contains an extremely high concentration of extracellular vesicles, which themselves contain immunomodulatory small RNAs and other molecules including cytokines (21–23). EV play a key role in immunosuppression in the cancer microenvironment, inducing tolerogenic phenotypes on local APCs which then influence the generation of regulatory or non-responsive T cells (57–59). In this study we fractionated cell-free semen into EV and EV-depleted (vesicle-depleted seminal plasma; VDSP) compartments, in order to better understand how each fraction impacts immunity in recipient cells.

We started by deriving dendritic cells (DCs) from peripheral blood monocytes, and exposing these cells to SEV and VDSP. Compared to untreated cells, only exposure to SEV increased CD141 and CD1a expression (**Figures 1A, B**). Immunoregulatory CD141+ DCs in the human dermis have shown lymph node migratory capacity, induced regulatory T cells and T cell hypo-responsiveness, and suppressed skin inflammation (60). CD1a+ APCs play a role in regulating T cell activation by presenting a variety of endogenous and exogenous lipid antigens to T cells (61). Upregulation of CD1a may be a consequence of exposure to the high lipid content of extracellular vesicles, as well as the lipid-rich nature of semen (62–64). CD1a+ DCs have also been reported to induce FOXP3 expression in activated T cells (65). In addition, we observed a significant upregulation of CD38 in SEV and VDSP treated cells. CD38 is a maturation marker for MoDCs (66). CD38 can inhibit the function of CD8+ T cells (67), facilitate the migration of mature DCs to the lymph nodes (68), and its high expression on myeloid-derived suppressor cells (MDSCs) correlates with more prominent immunosuppressive effects (69). Thus, it is plausible that semen components could direct the maturation and migration of DCs by upregulation of CD38 expression.

MoDCs generated in the presence of IL-10 have a well-described tolerance-inducing phenotype (70–73). We found that SEV and VDSP treatments induce a tolerogenic DC phenotype that is different from the one induced by IL-10. Interestingly, SEV and VDSP exposed cells demonstrated a more activated phenotype than IL-10 generated MoDCs. SEV and VDSP treated DCs maintained high expression levels of co-stimulatory CD86, and did not upregulate PD-L1 or TIM3 (**Figures 1J–L**). However, they did significantly upregulate ILT4 (**Figure 1H**), a receptor for immunomodulatory HLA-G, which is present in the non-cellular fraction of semen (74, 75). Ig-like transcripts (ILTs), including ILT3 and ILT4, are expressed on certain DCs, including in the decidua, and are associated with the induction of peripheral FOXP3+ Tregs (14, 70, 76–78). SEV and VDSP treated DCs also strongly upregulated the enzyme IDO (**Figure 1G**). Expression of IDO in tolerogenic APCs can suppress the proliferation and function of effector T cells, while promoting the generation of Tregs (79, 80). IDO initiates the oxidation of tryptophan, resulting in the production of kynurenine metabolites. An environment lacking tryptophan has been shown to cause human MoDCs to upregulate ILT3 and ILT4, enhancing their ability to induce Tregs (33). This indicates that low tryptophan levels caused by SEV- and VDSP-mediated induction of IDO activity could contribute to the upregulation of ILT4 expression by DCs.

In DCs emigrated out of vaginal tissue, we observed some similar phenotypic characteristics, mainly an increase in CD1a+ cells, high expression of co-stimulatory CD86 and CD80, and lack of upregulation of PD-L1 (**Figures 3**, **4**). The similarity of these results between monocyte-derived and tissue derived DCs suggests that the induction of a tolerogenic DC phenotype by SEV, and to a lesser extent by VDSP, occurs along common molecular pathways.

By quantifying the migration of DCs out of vaginal tissue following SEV or VDSP treatment *ex vivo*, we uncovered that SEV likely increase the migratory capacity of tissue DCs *in vivo* as well, suggesting that semen exposure enhances DC migration to the draining lymph nodes. In mice, DCs carrying allogeneic semen antigens and semen antigen-specific Tregs are found in the draining lymph nodes after mating (14, 81). We found that exposure to SEV, but not VDSP, significantly increased the migration of DCs, but not of other cell types (**Figure 5**). This suggests a model where both SEV and VDSP induce tolerance signals in DCs, but only SEV induce increased migration to lymph nodes to facilitate interaction with T cells and the generation of Tregs ready to support conception.

*In vivo*, migrating semen-exposed DCs engage with naïve T cells in the lymph nodes and shape the maturation of these T cells, setting the stage for the immune response or tolerance to the presented antigens. Co-cultures of SEV or VDSP-exposed MoDCs and vaginal emigrated cells with autologous naïve T cells resulted in the emergence of FOXP3+ Tregs co-expressing TIGIT in the semen exposed groups (**Figures 6**, **8**), suggesting a tolerogenic response to semen-derived antigens. TIGIT+ Tregs have a unique gene expression profile compared to TIGIT- Tregs and have more potent immunosuppressive capacities (82, 83). Many of these cells also expressed PD-1 and CTLA-4, which matches the high expression of immune regulatory molecules observed in decidual Tregs compared to blood Tregs (11, 14). We also observed increased expression of Helios in our co-cultured T cells (**Figures 7**, **9**). Helios was previously described as a marker for thymically derived, rather than peripherally induced Tregs (84), which would imply specificity for self-antigens rather than parental antigens. However, some peripherally induced Tregs do express Helios (85), and co-culture of CD4+ cells with fetal trophoblast cells or macrophages from the decidua increased both FOXP3 and Helios expression, implying the potential local induction of FOXP3+Helios+ cells (11). Here, we did not directly test whether our Helios+ cells resulted from proliferation of Helios+ cells in the initial pool, or from *de novo* expression of Helios, but this would be interesting to investigate in the future.

One of the limitations of this study is the use of a pooled semen sample from multiple donors to minimize interpersonal variability. We did observe variation according to recipient cells which is intriguing given the unknown etiology of many pregnancy complications. We speculate that variation in recipient response to tolerance induced by individual semen donors may be linked with risk of conditions like recurrent pregnancy loss, an area we are actively investigating. Such investigations could help uncover hidden factors contributing to infertility and facilitate the development of novel treatments or preventive strategies for infertility and pregnancy complications.

Previous reports have investigated how semen impacts DCs in humans (20, 53). In this study we investigated separately two bioactive components of semen – SEV and VDSP, in order to understand how each fraction affects DCs. We report that exposure to semen induces a unique phenotype in DCs, with upregulated potent immunosuppressive molecules (IDO, ILT4 or ILT3), while maintaining or even increasing co-stimulatory capacity (HLA, CD86 and CD80 expression). We propose a model whereby semen-exposed DCs are induced to migrate to the lymph nodes and present alloantigens, while inducing the differentiation of Tregs from naïve T cells. This would seed a pool of partner-specific Tregs poised to support implantation and pregnancy with the semi-allogeneic fetus, explaining the epidemiological findings of protection from preeclampsia with increased exposure to a specific partner’s semen (46–49, 51). Failure of this process could contribute to pregnancy complications such as recurrent pregnancy loss or preeclampsia, and a better understanding could lead to novel therapeutic strategies, including recommendation of seminal fluid priming (6, 86).

## Data Availability

The raw data supporting the conclusions of this article will be made available by the authors, without undue reservation.
