# Supplementary material for: Extracellular vesicles from human semen induce unique tolerogenic phenotypes in vaginal dendritic cells and regulatory T lymphocytes
Source: Front Immunol. 2025 May 12;16:1564002. doi: 10.3389/fimmu.2025.1564002 (PMC12104210; doi:10.3389/fimmu.2025.1564002)
Supplement: Supplementary file 1 [file DataSheet1.pdf]

Supplementary information

Extracellular vesicles from human semen induce unique tolerogenic phenotypes in dendritic cells and regulatory T cells

Shahrokh Paktinat[1], Michael G. Gravett[1], Cara Tobey[1], Anna Kirby[1], Whitney Horner[1], Rebecca Shaffer[1], Michael Fialkow[1], Nam Phuong Nguyen[2], Germán G. Gornalusse[1], Maryam Kalatehjari[1], Sean M. Hughes[1], Florian Hladik[1], Lucia Vojtech[1]*
[1]Department of Obstetrics and Gynecology, University of Washington, Seattle, WA 98195 USA
[2]Molecular Engineering and Sciences Institute, University of Washington, Seattle, WA 98195, USA
*Corresponding Author Email: luciav@uw.edu

Supplemental Table 1. 24-color flow cytometry panel used for characterization of antigen presenting cells (APCs) in monocyte-derived dendritic cell (MoDC) samples.

24c APC panel - OVERVIEW											
	Laser	Fluorophore	Antigen	Clone	Vendor	Cat#	RRID	Host	Compensation control	Cocktail	Titer
1	355nm	BUV395	CD40	5C3	BD Biosciences	565202	AB_2739110	Mouse	BD CompBead Plus Anti-Mouse Ig	Extracellular	1 to 80
2		BUV496	CD16	3G8	BD Biosciences	612945	AB_2870224	Mouse	BD CompBead Plus Anti-Mouse Ig	Extracellular	1 to 200
3		BUV563	CD56	NCAM16.2	BD Biosciences	612928	AB_2870213	Mouse	BD CompBeads Anti-Mouse Ig	Extracellular	1 to 160
4		BUV661	CD3	UCHT1	BD Biosciences	612964	AB_2870239	Mouse	BD CompBead Plus Anti-Mouse Ig	Extracellular	1 to 160
5		BUV737	CD86	FUN-1	BD Biosciences	612784	AB_2814790	Mouse	BD CompBead Plus Anti-Mouse Ig	Extracellular	1 to 160
6		BUV805	CD45	HI30	BD Biosciences	612891	AB_2870179	Mouse	BD CompBead Plus Anti-Mouse Ig	Extracellular	1 to 640
7	405nm	BV421	PD-L1	MIH1	BD Biosciences	563738	AB_2738396	Mouse	BD CompBeads Anti-Mouse Ig	Extracellular	1 to 160
8		Fixable Lime	Live/Dead	NA	Invitrogen	L34991	NA	NA	ArC™ Amine Reactive Beads	Live/Dead	1 to 500
9		BV570	CD14	M5E2	BioLegend	301832	AB_2563629	Mouse	BD CompBeads Anti-Mouse Ig	Extracellular	1 to 80
10		BV605	CD141	1A4	BD Biosciences	740421	AB_2740151	Mouse	BD CompBead Plus Anti-Mouse Ig	Extracellular	1 to 800
11		BV650	CD11b	D12	BD Biosciences	742640	AB_2740933	Mouse	BD CompBead Plus Anti-Mouse Ig	Extracellular	1 to 640
12		BV711	CD1a	SK9	BD Biosciences	745407	AB_2742961	Mouse	BD CompBead Plus Anti-Mouse Ig	Extracellular	1 to 160
13		BV786	CD85k (ILT3)	ZM3.8	BD Biosciences	742810	AB_2741062	Mouse	BD CompBead Plus Anti-Mouse Ig	Extracellular	1 to 160
14	488nm	BB515	TIM3	7D3	BD Biosciences	565568	AB_2744368	Mouse	BD CompBead Plus Anti-Mouse Ig	Extracellular	1 to 320
15		BB630	CD19	SJ25-C1	BD Biosciences	Custom		Mouse	BD CompBeads Anti-Mouse Ig	Extracellular	1 to 40
16		BB700	CCR7	3D12	BD Biosciences	566438	AB_2744306	Rat	BD™ CompBeads Anti-Rat and Anti-Hamster Ig	Extracellular	1 to 80
17		BB790	CD38	HIT2	BD Biosciences	Custom		Mouse	BD CompBead Plus Anti-Mouse Ig	Extracellular	1 to 80
18	532nm	PE	CD85d (ILT4)	42D1	BioLegend	338706	AB_2136524	Rat	BD™ CompBeads Anti-Rat and Anti-Hamster Ig	Extracellular	1 to 40
19		PE-CF594	CD163	GHI/61	BD Biosciences	562670	AB_2737711	Mouse	BD CompBeads Anti-Mouse Ig	Extracellular	1 to 40
20		PE-Cy5	CD80	L307.4	BD Biosciences	559370	AB_397239	Mouse	BD CompBead Plus Anti-Mouse Ig	Extracellular	1 to 20
21		PE-Cy7	IDO	eyedio	eBioscience	25-9477-42	AB_2637359	Mouse	BD CompBead Plus Anti-Mouse Ig	Intracellular	1 to 640
22	628nm	AF647	CD1c	F10/21A3	BD Biosciences	565048	AB_2744318	Mouse	BD CompBead Plus Anti-Mouse Ig	Extracellular	1 to 500
23		AF700	CD11c	B-Ly6	BD Biosciences	561352	AB_10612006	Mouse	BD CompBead Plus Anti-Mouse Ig	Extracellular	1 to 800
24		APC-H7	HLA-DR	G46-6	BD Biosciences	561358	AB_10611876	Mouse	BD CompBead Plus Anti-Mouse Ig	Extracellular	1 to 1600

Abbreviated staining procedure

1- Fc Receptor Blocking with BioLegend Human TruStain FcX (1:25) together with Live/Dead in DPBS for 20 min
2- Extracellular stain in BD Horizon™ Brilliant Stain Buffer Plus for 30 min
3- Fixation/Permeabilization in BD Cytofix/Cytoperm™ for 30 min
4- Intracellular stain in BD Perm/Wash™ Buffer for 30 min
5- Fixation in 4% Paraformaldehyde (PFA) for 10 min
6- Bring up in FACS Buffer (DPBS with 2% FBS)

Supplemental Table 2. 24-color flow cytometry panel used for characterization of vaginal tissue emigrated cells.

24c APC panel - OVERVIEW											
	**Laser**	**Fluorophore**	**Antigen**	**Clone**	**Vendor**	**Cat#**	**RRID**	**Host**	**Single stained control**	**Cocktail**	**Titer**
1	355nm	BUV395	CD40	5C3	BD Biosciences	565202	AB_2739110	Mouse	Invitrogen Ultracomp eBead Plus	Extracellular	1 to 80
2		BUV496	CD16	3G8	BD Biosciences	612945	AB_2870224	Mouse	Invitrogen Ultracomp eBead Plus	Extracellular	1 to 200
3		BUV563	CD56	NCAM16.2	BD Biosciences	612928	AB_2870213	Mouse	Cells	Extracellular	1 to 160
4		BUV661	CD3	UCHT1	BD Biosciences	612964	AB_2870239	Mouse	Cells	Extracellular	1 to 160
5		BUV737	CD86	FUN-1	BD Biosciences	612784	AB_2814790	Mouse	Invitrogen Ultracomp eBead Plus	Extracellular	1 to 160
6		BUV805	CD45	HI30	BD Biosciences	612891	AB_2870179	Mouse	Invitrogen Ultracomp eBead Plus	Extracellular	1 to 640
7	405nm	BV421	PD-L1	MIH1	BD Biosciences	563738	AB_2738396	Mouse	Invitrogen Ultracomp eBead Plus	Extracellular	1 to 160
8		Fixable Lime	Live/Dead	NA	Invitrogen	L34991	NA	NA	Cells	Live/Dead	1 to 500
9		BV570	CD14	M5E2	BioLegend	301832	AB_2563629	Mouse	Cells	Extracellular	1 to 80
10		BV605	CD141	1A4	BD Biosciences	740421	AB_2740151	Mouse	Invitrogen Ultracomp eBead Plus	Extracellular	1 to 800
11		BV650	CD11b	D12	BD Biosciences	742640	AB_2740933	Mouse	Invitrogen Ultracomp eBead Plus	Extracellular	1 to 640
12		BV711	CD1a	SK9	BD Biosciences	745407	AB_2742961	Mouse	Invitrogen Ultracomp eBead Plus	Extracellular	1 to 160
13		BV786	CD85k (ILT3)	ZM3.8	BD Biosciences	742810	AB_2741062	Mouse	Invitrogen Ultracomp eBead Plus	Extracellular	1 to 160
14	488nm	BB515	TIM3	7D3	BD Biosciences	565568	AB_2744368	Mouse	Invitrogen Ultracomp eBead Plus	Extracellular	1 to 320
15		BB630	CD19	SJ25-C1	BD Biosciences	Custom		Mouse	Invitrogen Ultracomp eBead Plus	Extracellular	1 to 40
16		BB700	CCR7	3D12	BD Biosciences	566438	AB_2744306	Rat	Invitrogen Ultracomp eBead Plus	Extracellular	1 to 80
17		BB790 or RB780	CD38	HIT2	BD Biosciences	Custom		Mouse	Cells	Extracellular	1 to 80
18	532nm	PE	CD85d (ILT4)	42D1	BioLegend	338706	AB_2136524	Rat	Invitrogen Ultracomp eBead Plus	Extracellular	1 to 40
19		PE-CF594	CD163	GHI/61	BD Biosciences	562670	AB_2737711	Mouse	Cells	Extracellular	1 to 40
20		PE-Cy5	CD80	L307.4	BD Biosciences	559370	AB_397239	Mouse	Invitrogen Ultracomp eBead Plus	Extracellular	1 to 20
21		PE-Cy7	IDO	eyedio	eBioscience	25-9477-42	AB_2637359	Mouse	Invitrogen Ultracomp eBead Plus	Intracellular	1 to 640
22	628nm	AF647	CD1c	F10/21A3	BD Biosciences	565048	AB_2744318	Mouse	Invitrogen Ultracomp eBead Plus	Extracellular	1 to 500
23		AF700	CD11c	B-Ly6	BD Biosciences	561352	AB_10612006	Mouse	Invitrogen Ultracomp eBead Plus	Extracellular	1 to 800
24		APC-H7	HLA-DR	G46-6	BD Biosciences	561358	AB_10611876	Mouse	Invitrogen Ultracomp eBead Plus	Extracellular	1 to 1600

Abbreviated staining procedure

1- Fc Receptor Blocking with BioLegend Human TruStain FcX (1:25) together with Live/Dead in DPBS for 20 min

2- Extracellular stain in BD Horizon™ Brilliant Stain Buffer Plus for 30 min

3- Fixation/Permeabilization in BD Cytofix/Cytoperm™ for 30 min

4- Intracellular stain in BD Perm/Wash™ Buffer for 30 min

5- Fixation in 4% Paraformaldehyde (PFA) for 10 min

6- Bring up in FACS Buffer (DPBS with 2% FBS)

Supplemental Table 3. 15-color flow cytometry panel used for characterization of CD4 T cells and identification of regulatory T cells (Tregs).

15c Treg panel - OVERVIEW

	Laser	Fluorophore	Antigen	Clone	Vendor	Cat#	RRID	Host	Single stained control	Cocktail	Titer
1	355nm	BUV395	LAG3	T47-530	BD Biosciences	569247	AB_3073773	Mouse	Invitrogen Ultracomp eBead Plus	Extracellular	1 to 40
2		BUV737	Ki-67	B56	BD Biosciences	567130	AB_2916461	Mouse	Invitrogen Ultracomp eBead Plus	Intracellular/intranuclear	1 to 320
3		BUV805	CD45	HI30	BD Biosciences	612891	AB_2870179	Mouse	Invitrogen Ultracomp eBead Plus	Extracellular	1 to 500
4	405nm	BV421	PD-1	EH12.1	BD Biosciences	565935	AB_2739399	Mouse	Invitrogen Ultracomp eBead Plus	Extracellular	1 to 160
5		BV570	CD4	RPA-T4	BioLegend	300533	AB_10896788	Mouse	Invitrogen Ultracomp eBead Plus	Extracellular	1 to 80
6		BV711	CTLA-4	BNI3	BioLegend	369631	AB_2892450	Mouse	Invitrogen Ultracomp eBead Plus	Intracellular/intranuclear	1 to 640
7	488nm	AF488	FOXP3	259D/C7	BD Biosciences	560887	AB_10562196	Mouse	Invitrogen Ultracomp eBead Plus	Intracellular/intranuclear	1 to 40
8		BB700	TIGIT	741182	BD Biosciences	747846	AB_2872309	Mouse	Invitrogen Ultracomp eBead Plus	Extracellular	1 to 320
9		RB780	CD25	2A3	BD Biosciences	568689		Mouse	Invitrogen Ultracomp eBead Plus	Extracellular	1 to 800
10	532nm	PE	Helios	22F6	BD Biosciences	563801	AB_2738428	Armenian Hamster	Invitrogen Ultracomp eBead Plus	Intracellular/intranuclear	1 to 640
11		PE-CF594	CD49b	12F1	BD Biosciences	564121	AB_2738606	Mouse	Invitrogen Ultracomp eBead Plus	Extracellular	1 to 320
12	628nm	AF647	CD127	HIL-7R-M21	BD Biosciences	560905	AB_647113	Mouse	Invitrogen Ultracomp eBead Plus	Extracellular	1 to 20
13		R718	IL10	JES3-19F1	BD Biosciences	567246		Rat	Invitrogen Ultracomp eBead Plus	Intracellular/intranuclear	1 to 160
14		APC-H7	CD3	SK7	BD Biosciences	560275	AB_1645476	Mouse	Invitrogen Ultracomp eBead Plus	Extracellular	1 to 160
15		Zombie NIR	Live/Dead	NA	BioLegend	423105	NA	NA	Cells	Live/Dead	1 to 1600

Abbreviated staining procedure

1- Fc Receptor Blocking with BioLegend Human TruStain FcX (1:40) together with Live/Dead in DPBS for 20 min
2- Extracellular stain in BD Horizon™ Brilliant Stain Buffer Plus for 30 min
3- Fixation/Permeabilization using eBioscience™ Foxp3 / Transcription Factor Staining Buffer Set for 40 min
4- Intracellular/intranuclear stain in Permeabilization Buffer for 40 min
5- Fixation in 4% Paraformaldehyde (PFA) for 10 min
6- Bring up in FACS Buffer (DPBS with 2% FBS)

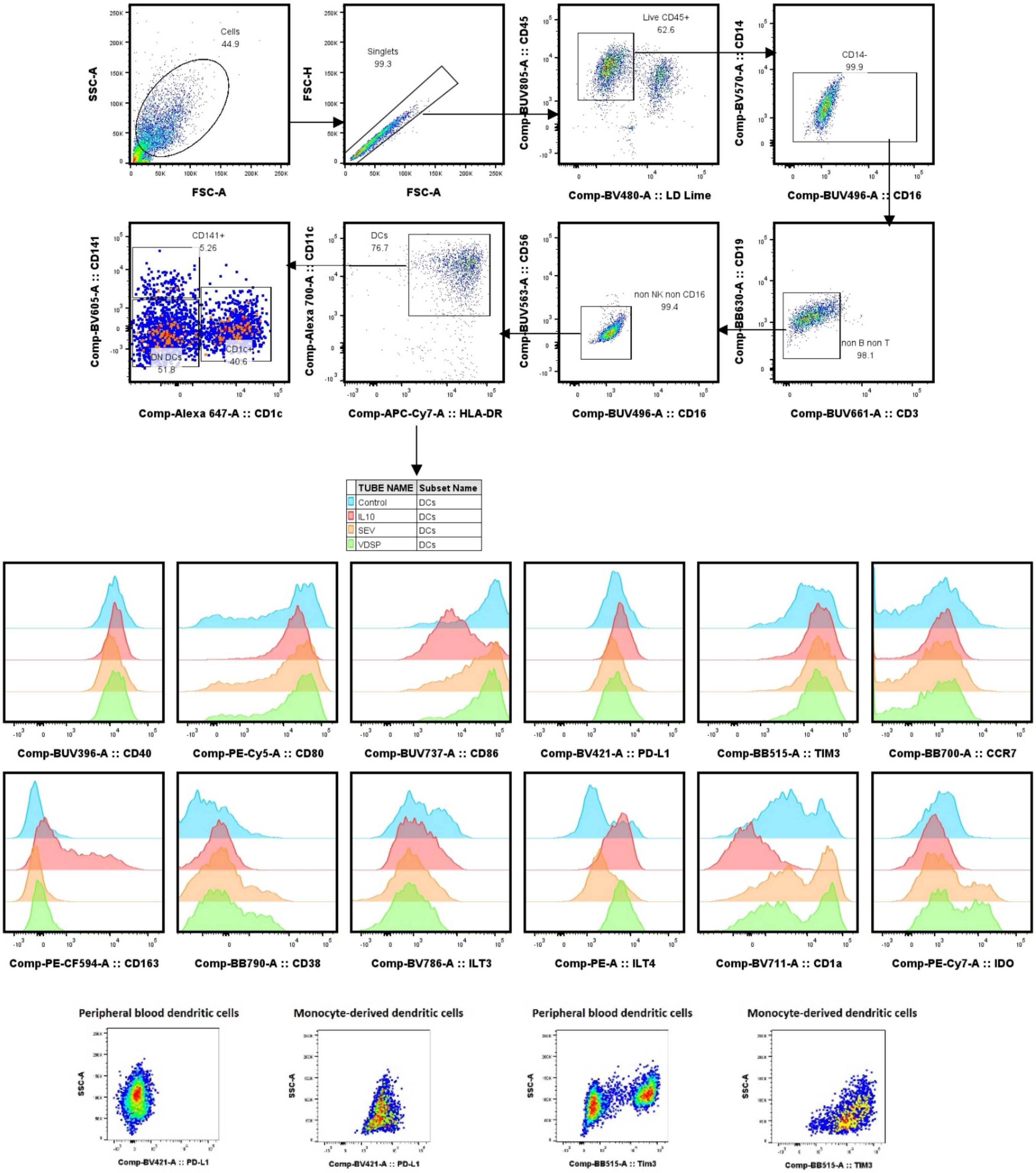

Supplemental Figure S1. Flow cytometry gating scheme for analyzing marker changes in monocyte-derived dendritic cells (MoDCs) after exposure to semen extracellular vesicles (SEV) or vesicle-depleted semen plasma (VDSP). IL-10-generated tolerogenic MoDCs and a group receiving DPBS served as the controls. DCs were defined as live CD45+ CD14- CD19- CD3- CD56- CD16- CD11c+ HLA-DR+ cells. Frequencies of CD141 and CD1c positive cells was determined. Further analysis was performed by gating on markers including CD40, CD80, CD86, PD-L1, TIM3, CCR7, CD163, CD38, ILT3, ILT4, CD1a, and indoleamine 2,3-dioxygenase (IDO). For markers with only one uniform positive population in MoDCs, we compared staining with peripheral blood DCs to assess staining specificity, as shown for PD-L1 and TIM3.

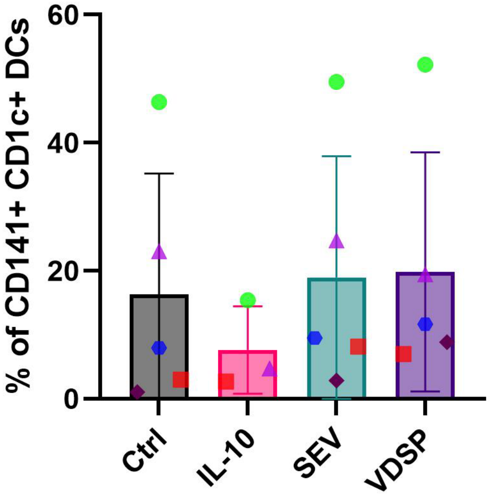

Supplemental Figure S2. Frequency of live CD45+ CD11c+ HLA-DR+ CD141+ CD1c+ monocyte-derived dendritic cells (MoDCs) induced by SEV. Each data point represents the average of two independent experiments conducted on the same donor on different days. Paired samples are represented by the same color/shape. Missing data points in the IL-10 group are due to insufficient cell numbers.

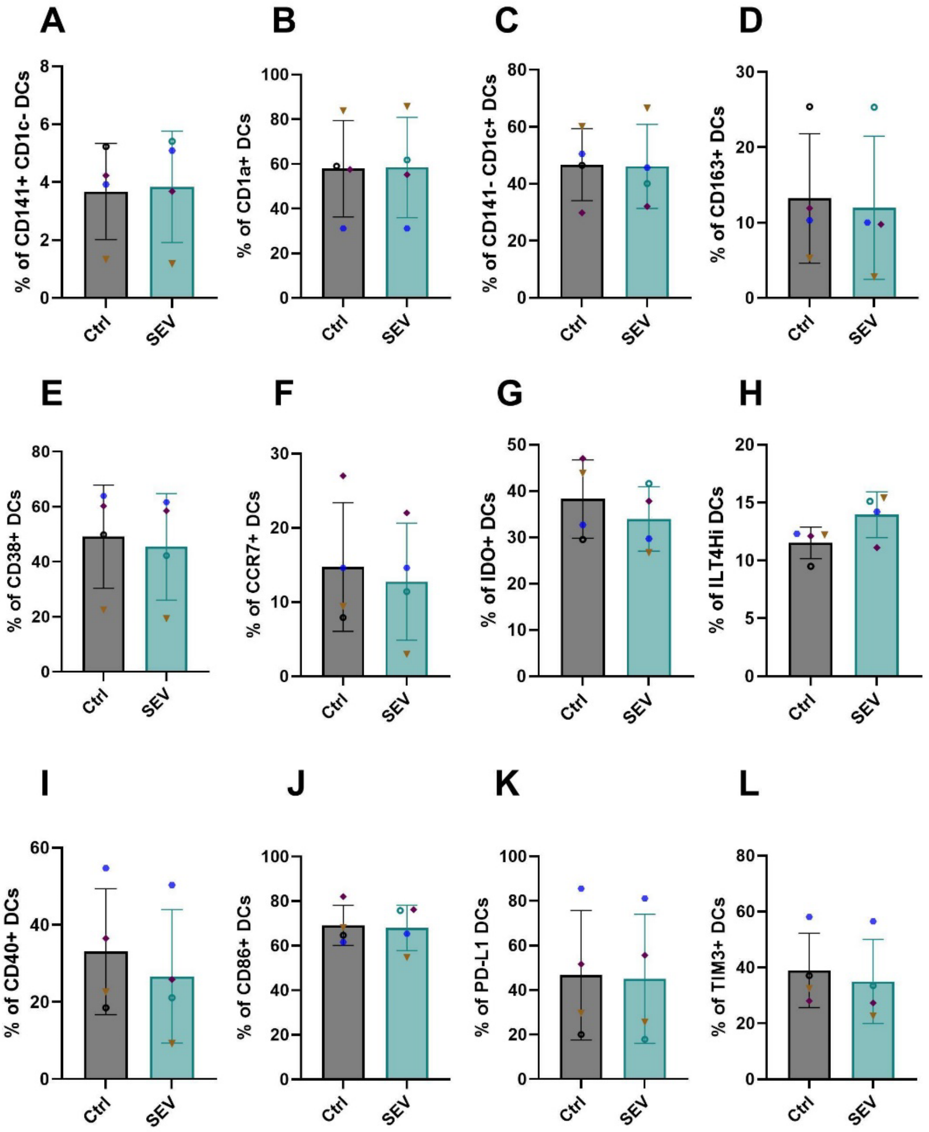

Supplemental Figure S3. Phenotypic changes induced by SEV on live CD45+ CD19- CD56- CD11c+ HLA-DR+ non-emigrated vaginal dendritic cells (DCs). Non-emigrated cells were harvested from the top chambers of the transwells; cells from top chambers exposed to VDSP were not harvested. (**A**) % CD141+ CD1c-, (**B**) % CD1a+, (**C**) % CD141- CD1c+, (**D**) % CD163+, (**E**) % CD38+, (**F**) % CCR7+, (**G**) % IDO+, (**H**) % ILT4Hi, (**I**) % CD40+, (**J**) % CD86+, (**K**) % PD-L1+, and (**L**) % TIM3+. Each data point represents a single donor. Paired samples are represented by the same color/shape.

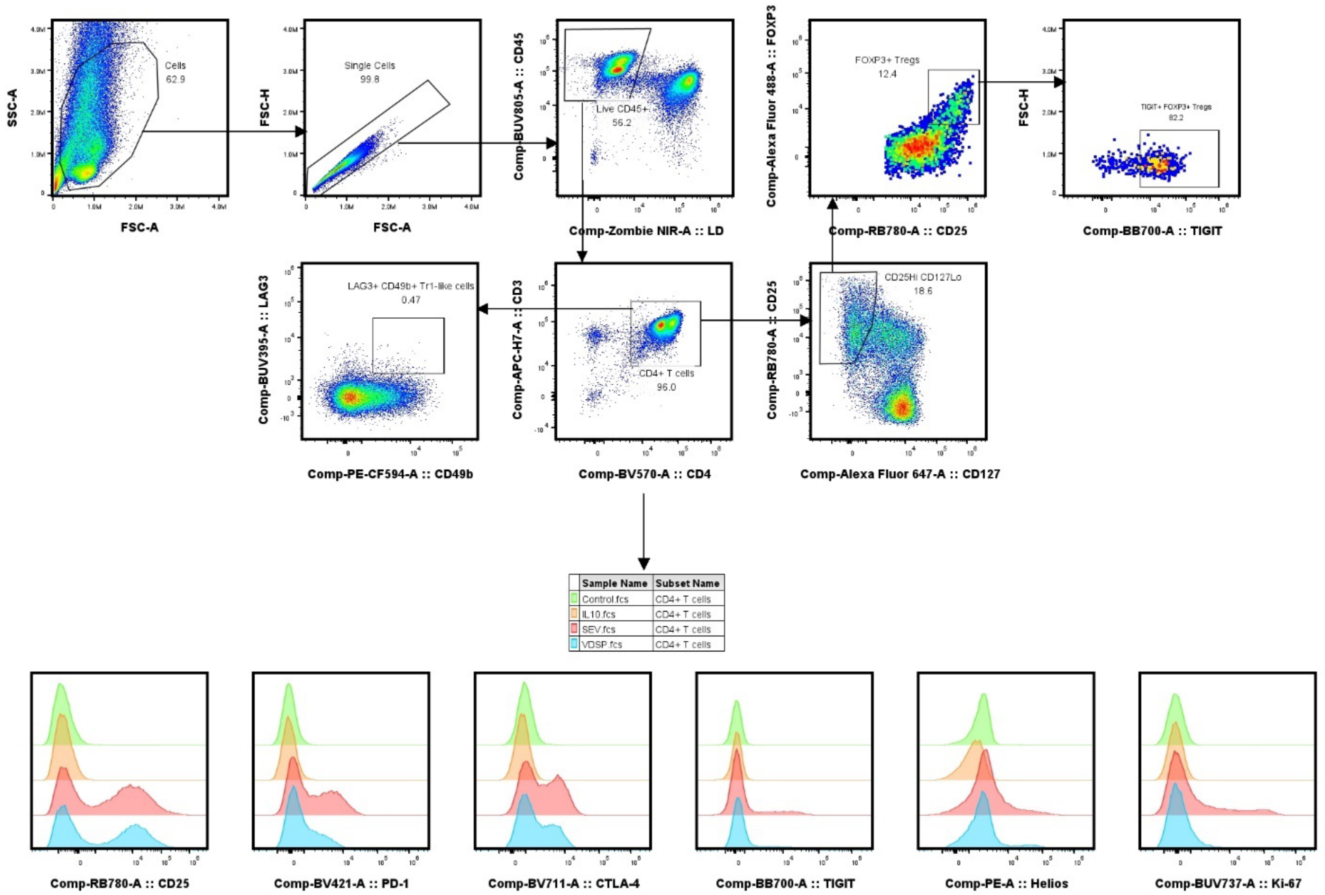

Supplemental Figure S4. Flow cytometry gating scheme for characterizing the CD4 T cells after co-culture with SEV- and VDSP-exposed monocyte-derived dendritic cells (MoDCs) or tissue emigrant cells. Three phenotypes of regulatory T cells (Tregs) were identified, including: live CD45+ CD3+ CD4+ CD25Hi CD127Lo FOXP3+ Tregs, live CD45+ CD3+ CD4+ CD25Hi CD127Lo FOXP3+ TIGIT+ Tregs, and live CD45+ CD3+ CD4+ LAG3+ CD49b+ Tr1-like Tregs. Further analysis of CD4 T cells was performed using activation and inhibitory markers including CD25, PD-1, CTLA-4, and TIGIT, as well as Treg stability marker Helios, and proliferation marker Ki-67.
